# A high-throughput 3D conjunctival spheroid model for standardized in vitro testing

**DOI:** 10.1038/s41598-026-63887-0

**Published:** 2026-07-23

**Authors:** Zhi Liang, Muhammad Aslam, Wahaj Ul Haq, Martina Wiesler, Su Naz Mutlu, Philipp Stahlhut, Raoul Verma-Fuehring, Ingrid Zahn, Friedrich P. Paulsen, Jürgen Groll, Jost Hillenkamp, Taufiq Ahmad, Malik Salman Haider

**Affiliations:** 1 Department of Ophthalmology, University Hospital Würzburg, Josef- Schneider Street 11, 97080 Würzburg, Germany; 2 Department of Functional Materials in Medicine and Dentistry, Institute of Functional Materials and Biofabrication (IFB), and Bavarian Polymer Institute (BPI), Julius-Maximilians-Universität Würzburg, 97070 Würzburg, Germany; 3 Institute of Functional and Clinical Anatomy, Friedrich-Alexander-University Erlangen-Nürnberg, Universitätsstr. 19, Erlangen, Germany

**Keywords:** New approach methodologies, Conjunctiva, Primary conjunctival spheroids, In vitro test system, Ocular surface, Ophthalmology, Biological techniques, Biotechnology, Cell biology, Materials science, Medical research

## Abstract

New Approach Methodologies (NAMs) based on human cells are increasingly needed to improve the physiological relevance, reproducibility, and ethical acceptability of preclinical ocular surface research. We developed a reproducible and scalable three-dimensional in vitro conjunctival spheroid model using primary human conjunctival epithelial cells and conjunctival fibroblasts as a human-relevant, scaffold-free test system. Agarose-based microwell arrays were fabricated via a combination of custom-made high-resolution 3D printing and polydimethylsiloxane (PDMS) replica molding, enabling the formation of uniform microwells suitable for spheroid culture and parallel production of size-controlled microtissues. Primary human conjunctival epithelial cells and fibroblasts were isolated from donor tissue obtained during routine ophthalmic surgeries and expanded under defined culture conditions. Fibroblast spheroids were first generated within agarose microwells at controlled seeding densities, resulting in stable and size-controlled aggregates. Subsequently, conjunctival epithelial cells were seeded onto pre-formed fibroblast spheroids to establish bilayered conjunctival spheroids that mimic native tissue organization. Spheroid development, morphology, and viability were systematically characterized using optical microscopy, live/dead assays, histological staining, immunofluorescence, and advanced imaging techniques including scanning electron microscopy (SEM), cryo-SEM, and transmission electron microscopy (TEM). Quantitative image analysis demonstrated consistent spheroid size and shape over time, while viability assays confirmed high cell survival. Histological and ultrastructural analyses revealed organized cellular architecture and extracellular matrix deposition, indicative of functional tissue-like constructs. By combining primary human cells, scaffold-free assembly, and microwell-based scalability, this platform contributes to the implementation of the 3Rs and provides a fit-for-purpose NAM for dry eye disease-related research, conjunctival inflammation studies, and early-stage preclinical compound testing.

## Introduction

The development of human-relevant New Approach Methodologies (NAMs) is a central objective in biomedical research, toxicology, and preclinical drug development^1,2^. In ophthalmology, there is a particular need for robust in vitro models that can reduce reliance on animal experimentation while improving the biological relevance and reproducibility of ocular surface studies^3^. The conjunctiva is a thin, transparent mucous membrane that plays a critical role in maintaining ocular surface homeostasis. It consists of a multilayered epithelium with integrated goblet cells and an underlying stromal matrix rich in fibroblasts, immune cells, and extracellular matrix (ECM) components, primarily collagen^4^. This structural composition contributes to tear film stability, ocular surface protection, and immune defense. However, disruptions in conjunctival integrity particularly goblet cell dysfunction and altered mucin production are associated with various ocular surface disorders, including dry eye disease (DED) and conjunctivitis etc^5,6^. DED is a prevalent, multifactorial condition, with prevalence estimates ranging from roughly 5% to 50%, and is characterized by tear film instability, hyperosmolarity, inflammation, and neurosensory dysfunction^7^. It leads to ocular discomfort, visual impairment, and, if untreated, long-term complications that significantly affect quality of life^8^.

3D in vitro test systems have emerged as essential tools for biomedical research, offering a more physiologically relevant alternative to traditional 2D cell cultures^9^. By better mimicking the native tissue architecture, cellular interactions, and ECM composition, these models provide deeper insights into disease mechanisms and therapeutic drug testing. Moreover, 3D models align with the 3Rs principles by reducing the need for animal testing while improving the reproducibility and translational potential of in vitro studies^10,11^. Therefore, human cell-based 3D models are increasingly recognized as fit-for-purpose NAMs for mechanistic studies, disease modelling, toxicity assessment, and preclinical compound screening^12^.

Despite the increasing demand for biologically relevant models, the development of sophisticated 3D conjunctival models remains limited. Schwebler et al. established a full-thickness 3D in vitro conjunctival model by co-culturing primary human conjunctival epithelial cells and fibroblasts on a rat tail collagen-based stromal equivalent, achieving robust goblet cell differentiation and a close resemblance to native tissue architecture^13^. Xie et al. developed a bi-layered tissue-engineered conjunctiva using a melt-electrowritten poly(ε-caprolactone) scaffold seeded with conjunctival stromal and epithelial cells^14^. Moving beyond synthetic scaffolds, Bannier-Hélaouët et al. generated human conjunctival organoids from adult stem cells to investigate ocular surface homeostasis and disease^15^, and Witt et al. proposed decellularized porcine conjunctiva repopulated with human conjunctival epithelial cells as an alternative substrate for ocular surface reconstruction^16^.

These models have been effective in addressing specific aspects of conjunctival research, including cell differentiation, inflammation studies, and applications with clinical relevance^13–16^. However, they are often technically complex, time-consuming, and require specialized materials or expertise, which limits their scalability and routine application. In addition, the reliance on animal-derived components such as rat tail collagen^13^, Matrigel^17^, and serum introduces variability, ethical concerns, and species-specific differences, reducing reproducibility and translational relevance. These limitations are particularly relevant from a NAM perspective, where standardization, transferability, reproducibility, and reduced dependence on animal-derived matrices are essential for broader implementation. Therefore, there remains a need for experimentally accessible, scalable, and human cell-based conjunctival models that can bridge the gap between simple 2D cultures and highly complex organoid or tissue-engineered systems.

Among the different approaches being explored for 3D in vitro model development, spheroids have also gained increasing attention as promising tools. These self-assembled, multicellular aggregates better simulate the 3D cellular organization and physiological properties of native tissues, making them valuable for studying cellular interactions, signaling pathways, and disease mechanisms^18^. Spheroids are particularly attractive as NAMs because they can be generated without permanent exogenous scaffolds, can be produced in multiwell-compatible formats, and allow standardized testing of multiple experimental conditions in parallel.

For ocular surface research, such features are highly relevant because they may enable mechanistic studies and early-stage compound testing using limited human donor material. However, despite their potential, spheroid-based models specifically addressing the human conjunctiva are still scarce. Previous studies by Fiorentzis et al.^19^ and Heinzelmann et al.^20^ generated 3D spheroids from conjunctival melanoma cell lines using commercially available, round-bottom ultra-low-attachment plates and employed these models to evaluate single or repetitive bleomycin-based electrochemotherapy studies. While these models highlight the feasibility of spheroid systems in ocular research, they are limited to cancer models and do not represent normal conjunctival physiology or inflammatory conditions such as DED.

Despite their advantages, spheroid models also present several limitations. Issues such as size inconsistency, difficulties in standardization and scalability and challenges in replicating the native ECM composition can affect reproducibility and biological interpretation^21,22^. Therefore, further optimization is required to fully exploit their potential as reliable and physiologically relevant in vitro models. Microwell-based spheroid production offers one strategy to address these limitations by improving control over initial cell aggregation, spheroid size, shape uniformity, and batch-to-batch reproducibility. In addition, sequential assembly of different cell types can support the generation of compartmentalized spheroids that better reflect native tissue organization.

In this context, biofabrication and tissue engineering approaches provide promising solutions by enabling precise control over cellular organization, tissue architecture and scalability. Building on our recent advancements in biofabrication technologies^23^, our study focuses on developing conjunctival spheroids as a scalable and reproducible in vitro test system. In contrast to existing complex 3D models^21,24^, our approach aims to combine biological relevance with experimental simplicity and scalability. To the best of our knowledge, this study is the first to demonstrate both the generation of conjunctival spheroids as a primary human cell based in vitro test system and their scalable production, neither of which has been reported previously. The intended context of use is early-stage ocular surface research, including DED-related disease modelling, conjunctival inflammation studies, and preclinical compound screening. While the model is not intended to fully replace the complexity of the native ocular surface, including tear film dynamics, immune cell recruitment, vascularization, innervation, and blinking-related mechanical stimulation, it provides a standardized and experimentally accessible human in vitro platform for defined applications. By combining primary human cells, scaffold-free spheroid formation, and scalable microwell-based production, this platform contributes to the development of human-relevant NAMs in ophthalmology and supports the implementation of reduction and partial replacement strategies in ocular surface research.

## Materials and methods

### Preparation of agarose microwells

#### 3D printing of positive mold micropillars

To fabricate the microwells, master molds were first produced by 3D printing with a Prusa SL1S DLP printer (Prusa Research, Prague, Czech Republic) using FotoDent resin (Dentamid Fotodent 405 nm, Dreve, Dresden, Germany). Polydimethylsiloxane (PDMS) (Sylgard 184 Silicone Elastomer, Dow, Midland, MI, USA) was then cast onto the 3D-printed molds to create a PDMS stamp featuring micropillars. After curing, this PDMS stamp was used to transfer the honeycomb-inspired microwell pattern into agarose.

#### Fabrication of negative agarose mold for cell culture

The 3D printed micropillars were sterilized by immersion in 70% ethanol for two hours, then rinsed three times with sterile phosphate-buffered saline (PBS). Under sterile conditions, the micropillar was positioned tip-up in the center of a 12-well plate (Corning Incorporated, One Riverfront Plaza, Corning, NY 14831 USA) using sterile tweezers. A 2% agarose solution was prepared by dissolving 2 g of agarose powder (peqGOLD Agarose, universal, VWR Life Science, 2023) in 100 mL of sterile distilled water. The solution was then heated in a microwave at 800 W for 3 min until it became clear. After heating, the solution was transferred to the sterile hood, and 6 mL of the solution was added to each well. The agarose was allowed to solidify for 30 min. Once solidified, the negative agarose mold was carefully removed from the well plate by inserting a double-ended spatula vertically along the well wall. The micropillar was gently pressed with tweezers to ensure detachment of the agarose, after which it was removed vertically. Two vertical channels were created along the exterior of the agarose mold using a hollow punch (BOEHM, JLB 320CM, 2023, France) to facilitate culture medium exchange, and to improve the handling and placement of the molds in 12-well plates, where they were arranged symmetrically. The microwells themselves were circular with a rounded bottom to promote uniform cell aggregation, whereas their arrangement followed a honeycomb packing pattern. This design maximized the density of microwells within the available culture area, thereby minimizing unused surface between adjacent wells and reducing the likelihood of cells settling outside the microwells during seeding.

#### Sterilization of negative agarose molds

For sterilization, 0.8 mL of sterile PBS was dispensed into each agarose well of a 12-well plate and subjected to UV irradiation overnight. The following day, the plate lid was removed, the PBS was siphoned, and the negative agarose mold underwent an additional UV irradiation for 1 h. Subsequently, fibroblast basal medium DMEM ((Thermo Fisher, Waltham, USA) supplemented with 10% fetal calf serum (FCS, Bio&SELL GmbH, Feucht, Germany) and 1% Penicillin/Streptomycin (Sigma-Aldrich, Darmstadt, Germany) was added to the wells for a 30-minute incubation, after which it was removed. The wells were ready for subsequent cell culture.

### Isolation and culture of primary conjunctival fibroblast and epithelial cells

Residual human conjunctival tissue was obtained predominantly from patients undergoing routine 20-gauge pars plana vitrectomy at the Department of Ophthalmology (Augenklinik und Poliklinik), University Hospital Würzburg (Universitätsklinikum Würzburg AöR), Würzburg, Germany. Only macroscopically normal conjunctival tissue without evidence of conjunctival inflammation, scarring, or other ocular surface pathology was used for cell isolation. Written informed consent was obtained from all donors prior to tissue collection. The tissues were collected in accordance with approval from the local ethics committee (Medizinische Ethikkommission an der Julius-Maximilians-Universität Würzburg; reference number 2025 − 540_1-dvhb; Würzburg, 16 January 2026) and processed in compliance with the Declaration of Helsinki and applicable local regulations for research on human material.

Human donor conjunctival tissue was obtained and stored in DMEM until further processing. The cells were isolated according to our previously reported method^25^. Briefly, the tissue was incubated with dispase solution (2 U/mL) for 1 h at 37 °C. The epithelial layers were carefully scraped using curved tweezers and transferred to a Petri dish containing conjunctival epithelial cell basal medium (named as P1: Keratinocyte Growth Medium2 + SupplementMix (KGM-2, Promocell, Catalog No. C-20011)). The epithelial cells in P1 medium were then subjected to centrifugation at 300×g for 5 min at room temperature (RT), and the supernatant was discarded. The resultant cell pellet was resuspended in 2 mL of P1 medium and transferred to a T25 culture flask for subsequent expansion. On day 2, the medium was supplemented with 1–2 mL of fresh P1 medium. By day 3, once cell attachment was observed, the medium was replaced with 5 mL of fresh P1 medium.

To isolate conjunctival fibroblasts, the remaining stromal tissue, (after mechanical scraping of the epithelial layer), was digested with collagenase A solution (5 U/mL) at 37 °C for 1 h. After enzymatic digestion, the tube was centrifuged at 300×g for 5 min at RT, and the resulting pellet was resuspended in 3 mL of DMEM. The fibroblast suspension was then seeded into a T25 culture flask. For both epithelial cells and fibroblasts, the culture medium was replaced every second day. Epithelial cells were maintained in 5 mL of P1 medium, while fibroblasts were cultured in 5 mL of DMEM.

### Biofabrication of fibroblast spheroids

In this study, primary human conjunctival fibroblasts from passage 6–12 were used. The cells were seeded into the negative agarose molds at five different densities of approximately 1,000, 2,000, 3,000, 4,000, and 5,000 cell per microwell. The fibroblast cell suspension with 800 µL volume was introduced into each agarose mold in a clockwise direction, starting from the outer area and moving toward the inner area. The agarose molds containing the cell suspension were then centrifuged (ROTINA 380R, Hettich, Tuttlingen, Germany) at 1200 rpm for 5 min to ensure uniform distribution and cell sedimentation. DMEM medium was added to the side channels to establish the culture environment, and the medium was replaced every 48 h to maintain optimal growth conditions.

### Biofabrication of conjunctival spheroid via epithelial cell seeding

When the size of the fibroblast spheroids became stable on day 5, human primary conjunctival epithelial cells (passage 5–8) were seeded into the agarose molds containing fibroblast spheroids at a density of 1500 epithelial cells per microwell.

Initially, the epithelial cells were harvested into the proliferation medium (P2: P1 + 0.48% 300mM CaCl2-Solution) and prepared as a cell suspension. Next, the DMEM was removed from the fibroblast spheroid agarose mold, and the epithelial cell suspension was carefully added, starting with the outermost ring of agarose mold and then proceeding clockwise toward the center. Afterward, the culture medium in the side channels was replaced with P2 medium. The system was then placed on a shaker (Titramax 100; Heidolph, Schwabach, Germany) at 350 rpm and incubated in a 37 °C, 5% CO₂ incubator (HERAcell 150i; Thermo Scientific, Heraeus, Germany) overnight. During this period, essentially all epithelial cells attached to the fibroblast spheroids, forming stable bilayer co-culture spheroids. The shaking was stopped the following day, and the medium was replaced every 48 h thereafter.

### Characterization of spheroid

#### Size monitoring of fibroblast and conjunctival spheroids over time

Agarose molds containing spheroids were placed under an optical microscope (ECLIPSE Ts2R; Nikon, Tokyo, Japan) for continuous observation of size changes following cell seeding. Day 1 was defined as 24 h after cell seeding. Images of fibroblast spheroids were captured on days 1, 3, 5, 7, 9 and 11, while images of conjunctival spheroids were captured on days 1, 3, 5, 7 and 9. For each well, spheroids within 80 randomly selected microwells were photographed, and the Aspect ratio (Feret/MinFeret) and area of the spheroids were measured using Fiji (ImageJ2, version 2.16.0/1.54 g; http://imagej.net). The average values were then calculated for further analysis, and the data are presented as mean ± standard deviation (SD).

#### Live/Dead assay

For Live/Dead staining, day 10 fibroblast spheroids were first washed with sterile 1 x PBS and then incubated with a working solution of Live Cell Staining Dye (calcein acetoxymethyl ester, Calcein AM) and Dead Cell Staining Dye (ethidium homodimer) prepared from the assay kit (ab287858; Abcam, Cambridge, UK). The solution was mixed in a ratio of 1:5 (2.4 µL of Live Cell Staining Dye and 1.2 µL of Dead Cell Staining Dye in 6 mL of Assay Buffer). The dye was added to the wells (500 µL/well for 12-well plates), ensuring complete submersion of the spheroids. After incubation for 15 min at 37 °C in the dark, spheroids were analyzed under a light and fluorescence microscope (ECLIPSE Ts2R; Nikon, Tokyo, Japan). Images were subsequently processed using Fiji.

#### Cryo-sectioning and immunostaining of spheroids

To examine the cross-sectional morphology, spheroids were first fixed in 4% paraformaldehyde at RT for 3 h. After fixation, a 1% (w/v) agarose solution was prepared, and once the solution had cooled to approximately 37 °C while remaining in a liquid state, the spheroids were embedded in the agarose. The agarose-embedded spheroids were then placed into OCT solution (Sakura Finetek, Torrance, CA, USA) overnight. On the following day, the samples were frozen in liquid nitrogen and stored in a -80 °C freezer overnight. Subsequently, approximately 10 μm thick sections of the spheroids were prepared using a Cryostat Cryocut microtome (CM1510 S, Leica Microsystems, Wetzlar, Germany) and stored at -20 °C for subsequent staining and observation.

Cryo-sections of spheroids were first washed in distilled water for 10 min, followed by permeabilization with 0.1% Triton X-100 at RT for 10 min, and subsequently blocked with blocking buffer (2% normal goat serum in PBS containing 0.1% Triton X-100) for 1 h at RT. The sections were then incubated with primary antibodies against vimentin (1:250), CK19 (1:100), collagen I (1:300) and collagen IV (1:300) overnight at 4 °C. The following day, samples were washed twice with PBS for 5 min each. Following primary antibody incubation, the samples were treated with anti-mouse or anti-rabbit secondary antibody (1:500) for 1 h at RT. Nuclei were counterstained with Hoechst 33,258 (1:2000), and F-actin was stained with rhodamine-phalloidin (1:500). Finally, the stained samples were mounted and observed under Keyence fluorescence microscope (BZ-X810; Keyence Corp., Osaka, Japan).

#### Hematoxylin and eosin (H&E) staining of paraffin-sections

H&E staining was performed to assess the cellular distribution within fibroblast and conjunctival spheroids. The spheroids were harvested and embedded in Histogel (Epredia, HG-4000-012) to facilitate handling during subsequent histological processing. The Histogel-embedded samples were placed into tissue cassettes, dehydrated through a graded ethanol series, cleared in chloroform (Sigma-Aldrich, 32211–2.5 L-M), and infiltrated with molten paraffin. Samples were then embedded in paraffin using an embedding station (MEDITE, TES 99) to generate paraffin blocks. Paraffin blocks were sectioned at 5 μm thickness using a rotary microtome (LEICA SM 2000R), and sections were mounted onto microscope slides for further staining or analysis.

#### Morphological characterization of fibroblast and conjunctival spheroids by scanning electron microscopy (SEM), Cryo-SEM and transmission electron microscopy (TEM)

##### Scanning electron microscopy (SEM)

The fibroblast (day 5) and conjunctival (day 10) spheroids were first washed 3 times with PBS in the culture dish. Following this, they were fixed with 6% glutaraldehyde (Sigma-Aldrich, St. Louis, MO, USA; Cat. No. 340855) (prepared by diluting 25% glutaraldehyde in 1x PBS) and incubated on ice for 15 min. Dehydration was performed in a graded series of solutions, with PBS washes conducted on ice and ethanol dehydration steps carried out at RT. The dehydration series included sequential incubations in 70%, 90%, and 100% ethanol, each for 10 min. After dehydration, the samples were dried by immersing in hexamethyldisilazane (HMDS; Sigma-Aldrich, St. Louis, MO, USA; Cat. No. 440191) for two 15-minute treatments, followed by air drying. The spheroids were secured onto carbon conductive adhesive tape and placed onto an SEM specimen holder for imaging using SEM (S-2380 N, Hitachi, Tokyo, Japan). The SEM images with focus ion beam cutting (FIB-SEM) were taken with a Zeiss Crossbeam 340 Field-Emission Electron Microscope (Zeiss Microscopy, Oberkochen, Germany) at an acceleration voltage of 2 kV. A Ga-FIB with an acceleration voltage of 30 kV and a beam current of 1,5nA was used to cut the samples.

##### Cryo-scanning electron microscopy (Cryo-SEM)

For Cryo-SEM, the fibroblast and conjunctival spheroids were harvested and gently rinsed three times with PBS. The specimens were immobilized by sandwiching them between two aluminum carriers (3 mm diameter) featuring a 2 mm recess for positioning and were immediately vitrified by plunge-freezing in slushed nitrogen (-210 ℃). Thereafter, the frozen samples were handled exclusively under high vacuum (< 1 × 10^− 3^ mbar) and cryogenic conditions and transferred using a Leica EM VCT100 Cryo Shuttle (Leica Microsystems, Wetzlar, Germany). To expose an unprocessed surface for inspection, one carrier was removed at -140 °C to induce fracture and reveal a fresh spheroid face. The exposed surface was then cryo-etched by controlled sublimation for 15 min at -85 ℃ in a Leica EM ACE600 sputter coating unit (Leica Microsystems, Wetzlar, Germany). Next, the samples were sputter-coated with a 2.5 nm platinum layer and transferred into the SEM chamber (Crossbeam 340). Imaging was performed on a cryo-stage at -156 ℃ using an acceleration voltage of 8 kV.

##### Transmission electron microscopy (TEM)

For TEM, spheroids were harvested and fixed in ITO solution (2.5% paraformaldehyde, 2.5% glutaraldehyde in 0.1 M cacodylate buffer). Samples were subsequently pre-embedded in HistoGel™ (Epredia™, Portsmouth, NH, USA), processed according to standard TEM tissue preparation procedures, and embedded in Epon resin. Ultrathin sections were prepared and examined using a JEM-1400Plus transmission electron microscope (JEOL, Tokyo, Japan).

## Results and discussion

**Fig. 1:**
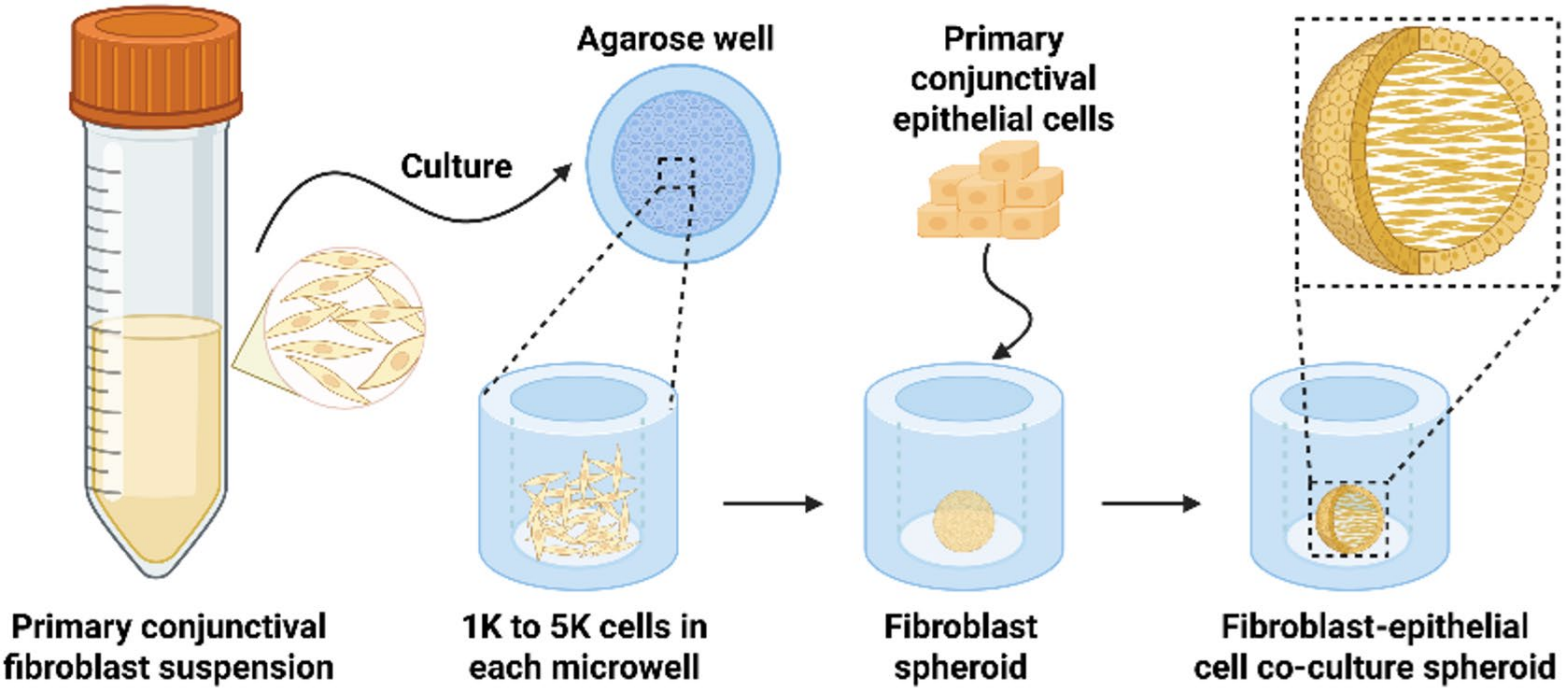
Schematic representation of conjunctival spheroids formation in agarose molds. Created in BioRender.

To establish a 3D conjunctival spheroid model, we first isolated primary conjunctival fibroblasts and epithelial cells and generated single-cell suspensions as previously reported^25^. Fibroblasts were seeded into negative agarose molds, where they aggregated to form stable fibroblast spheroids. Once the spheroids reached a compact and uniform morphology, conjunctival epithelial cells were subsequently seeded onto their surface, allowing the epithelial layer to adhere and organize around the fibroblast core. This sequential assembly yielded a well-defined bilayered conjunctival spheroid structure (Fig. 1).

Primary conjunctival fibroblasts were first seeded into agarose negative molds at densities ranging from 1 K to 5 K cells per microwell to generate fibroblast spheroids. Subsequently, at day 5 primary conjunctival epithelial cells (1.5 K cells per microwell) were overlaid onto the fibroblast spheroids. The agarose molds were placed on an orbital shaker at 300 rpm in a 37 °C incubator overnight, during which epithelial cells gradually adhered to and enveloped the fibroblast spheroids, resulting in the formation of stable bilayer conjunctival spheroids.

**Fig. 2:**
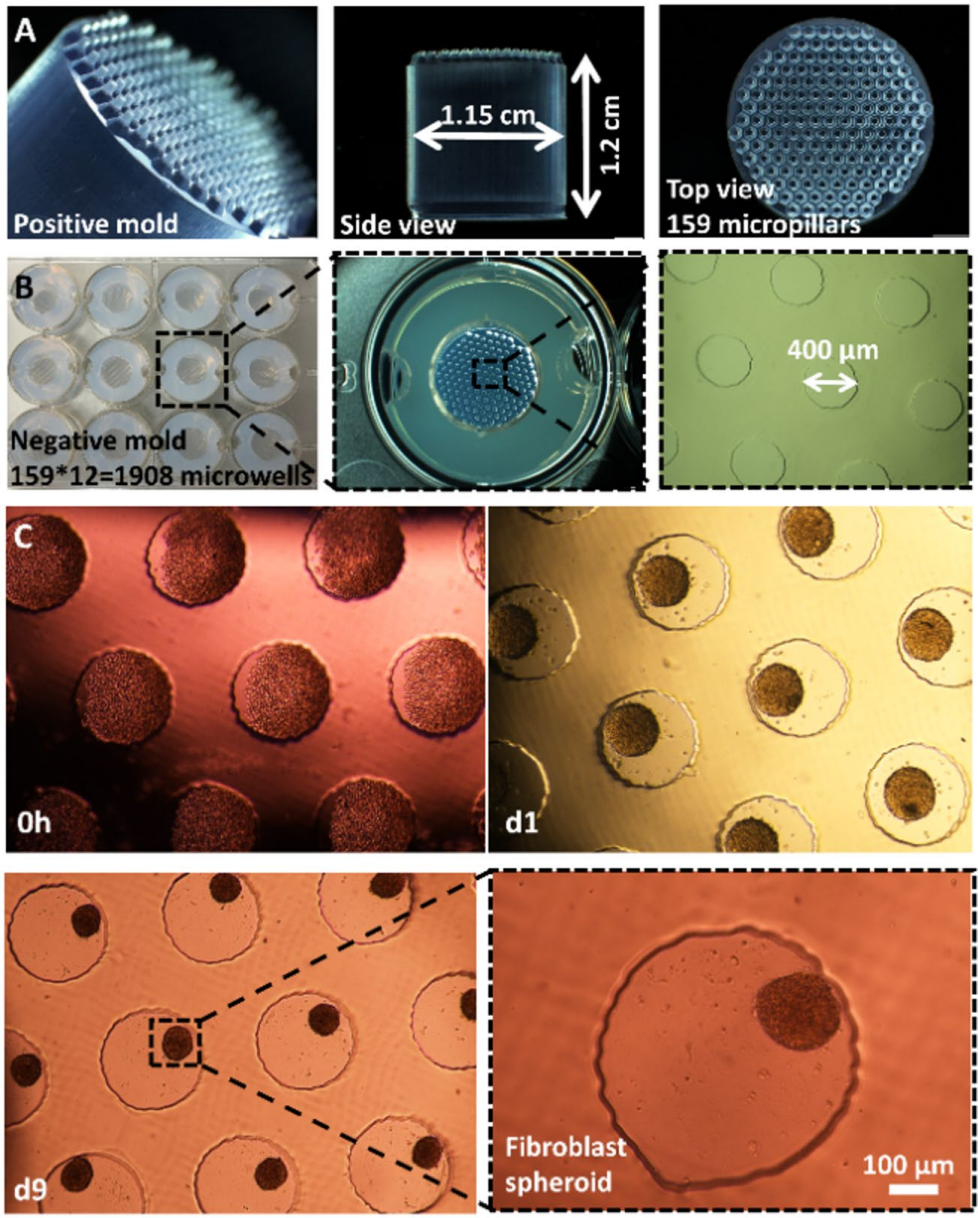
Fabrication of agarose molds and formation of fibroblast spheroids. (**A**) Design of the 3D-printed positive mold (1.2 cm diameter) containing 159 micropillars shown in oblique, side, and top views. (**B**) Generation of the agarose negative mold by casting 2% agarose solution over the positive mold. Each mold consisted of 159 microwells, and 12 molds yielded a total of 1908 microwells. Representative images show the molded agarose in a culture plate and the uniform microwell geometry. (**C**) Optical images showing formation of fibroblast spheroids within the microwell array (3 K cells per microwell). Uniform-sized spheroids formed by day 1 and continued to compact from day 1 to day 9. A magnified view shows a representative spheroid.

Following the overall workflow, in parallel, we fabricated an agarose microwell system to generate uniform fibroblast spheroids (Fig. 2). A positive mold was developed by 3D printing, forming a 1.15-cm-diameter, 1.2-cm-high cylindrical structure containing 159 Y-shaped micropillars at its apical surface (Fig. 2A). The Y-shaped micropillars facilitated consistent microwell geometry. Using a 12-well plate with one mold placed in each well, and with each mold containing 159 micropillars, a total of 1,908 agarose microwells (159 × 12) can be produced simultaneously (Fig. 2B). Using this negative agarose microwell system, 3,000 primary conjunctival fibroblasts were seeded into each well. The cells rapidly aggregated upon seeding, forming compact and increasingly defined spheroids by day 1, which further stabilized and condensed by day 9 (Fig. 2C). These fibroblast spheroids served as the stromal core for the subsequent epithelial cells seeding step to construct the bilayered conjunctival spheroids.

**Fig. 3:**
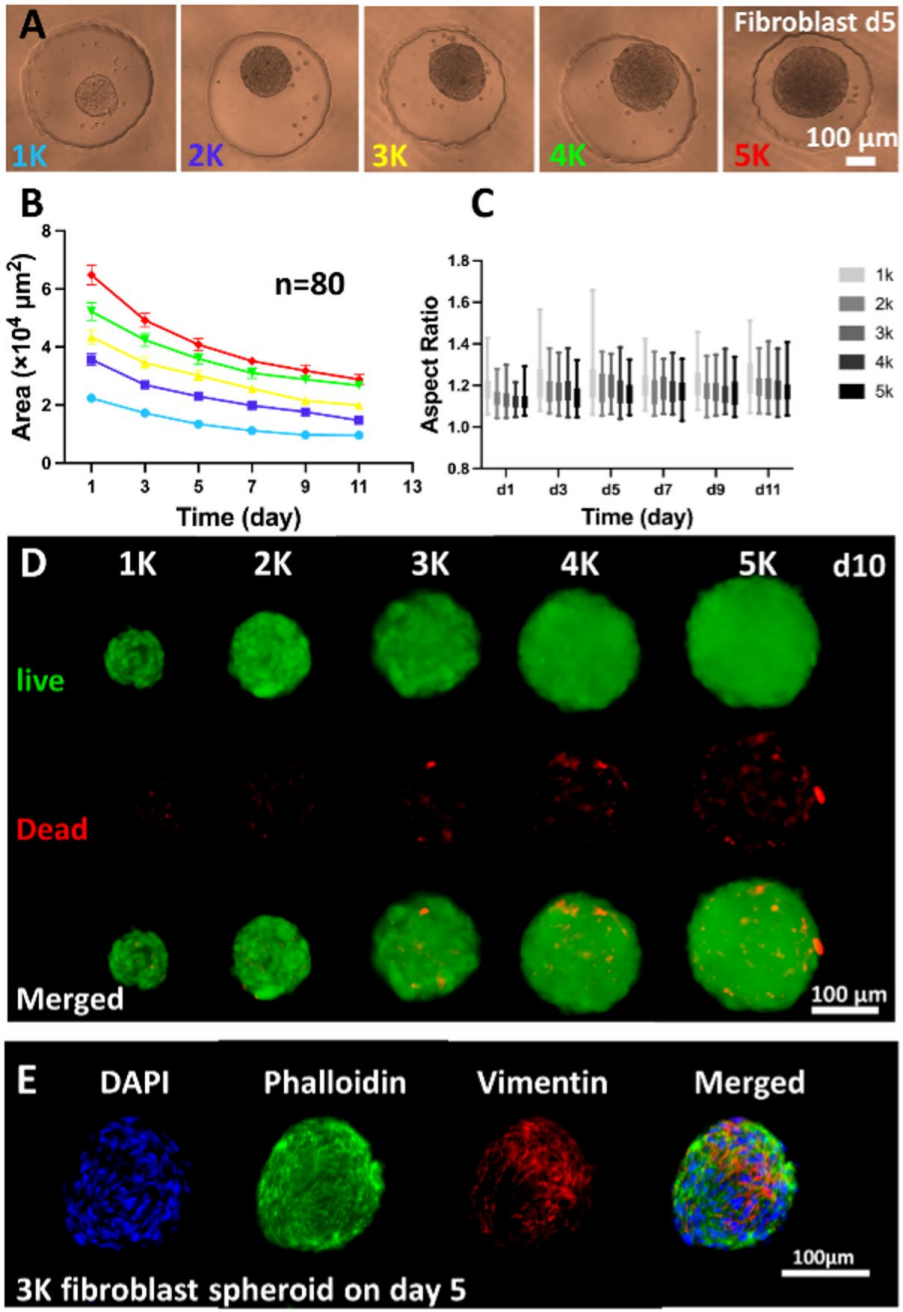
Assessment of fibroblast spheroid shape, size, viability, and marker expression. (**A**) Optical images of fibroblast spheroids generated from 1 K to 5 K cells demonstrate a clear size dependence based on the initial cell number. (**B**) Spheroid projected area (µm^2^) was quantified from brightfield images using ImageJ (*n* = 80). At day 1, mean spheroid area increased with seeding density, ranging from (2.2 ± 0.1) × 10^4^ µm^2^ (1 K) to (6.5 ± 0.3) × 10^4^ µm^2^ (5 K). Spheroid area decreased over time and reached stable values by day 11, ranging from (1.0 ± 0.1) × 10^4^ µm^2^ to (2.9 ± 0.2) × 10^4^ µm^2^. Data are presented as mean ± SD. (**C**) Aspect ratio (Feret/MinFeret) was measured in ImageJ (*n* = 80). Across all seeding densities, aspect ratios remained stable over time, with mean values ranging from 1.125 to 1.230 and mean ± SD values below 1.5 at all the time points. (**D**) Live/dead staining performed on day 10. (**E**) IF of 3 K spheroids at day 5 shows preserved nuclear architecture (DAPI), cytoskeletal organization (phalloidin), and fibroblast-specific marker expression (vimentin).

To optimize the size and structural quality of fibroblast spheroids, we seeded 1 K to 5 K conjunctival fibroblasts into each microwell. Optical microscopy at day 5 revealed a clear cell number dependent increase in spheroid diameter across the five seeding densities (Fig. 3A). Quantitative ImageJ analysis (area measurement) further confirmed this trend: at day 1, spheroids generated from 1 K, 2 K, 3 K, 4K, and 5 K cells exhibited mean areas of (2.2 ± 0.1) × 10^4^ μm², 3.6 ± 0.2 × 10^4^ μm², 4.3 ± 0.2 × 10^4^ μm², 5.2 ± 0.3 × 10^4^ μm², and 6.5 ± 0.3 × 10^4^ μm², respectively. The spheroids progressively reorganized and compacted over time, with areas decreasing to 1.3 ± 0.1 × 10^4^ μm² (1 K), 2.3 ± 0.1 × 10^4^ μm² (2 K), 3.0 ± 0.2 × 10^4^ μm² (3 K), 3.6 ± 0.2 × 10^4^ μm² (4K), and 4.0 ± 0.2 × 10^4^ μm² (5 K) by day 5. From day 7 onward, compaction plateaued, with only minor changes observed through day 9. By day 11, spheroid areas had stabilized at 1.0 ± 0.1 × 10⁴ µm², 1.5 ± 0.1 × 10⁴ µm², 2.0 ± 0.1 × 10⁴ µm², 2.7 ± 0.1 × 10⁴ µm², and 2.9 ± 0.2 × 10⁴ µm², respectively (Fig. 3B). To evaluate spheroid morphology, aspect ratio was measured as an indicator of roundness. All groups-maintained aspect ratios close to 1 throughout the observation period of 11 days, with values consistently below 1.5, demonstrating robust spherical morphology and structural stability across different seeding densities (Fig. 3C). Cell viability, assessed by live/dead staining on day 10, demonstrated high overall viability across all spheroid sizes (Fig. 3D). As expected, larger spheroids (4–5 K cells) exhibited a slightly higher number of dead cells than smaller spheroids (1–3 K cells), consistent with the nutrient diffusion limitation. Considering spheroid size, compaction behavior, structural uniformity, and viability, 3 K spheroids at day 5 were selected as the optimal condition for generating the subsequent bilayered conjunctival spheroids. Immunofluorescence staining of 3 K fibroblast spheroids on day 5 revealed dense nuclear packing (DAPI), well-organized cortical actin (phalloidin), and a robust vimentin intermediate filament network, characteristic of fibroblasts (Fig. 3E). These features confirm that the spheroids possess a mature, compact architecture suitable for epithelial cell seeding.

**Fig. 4:**
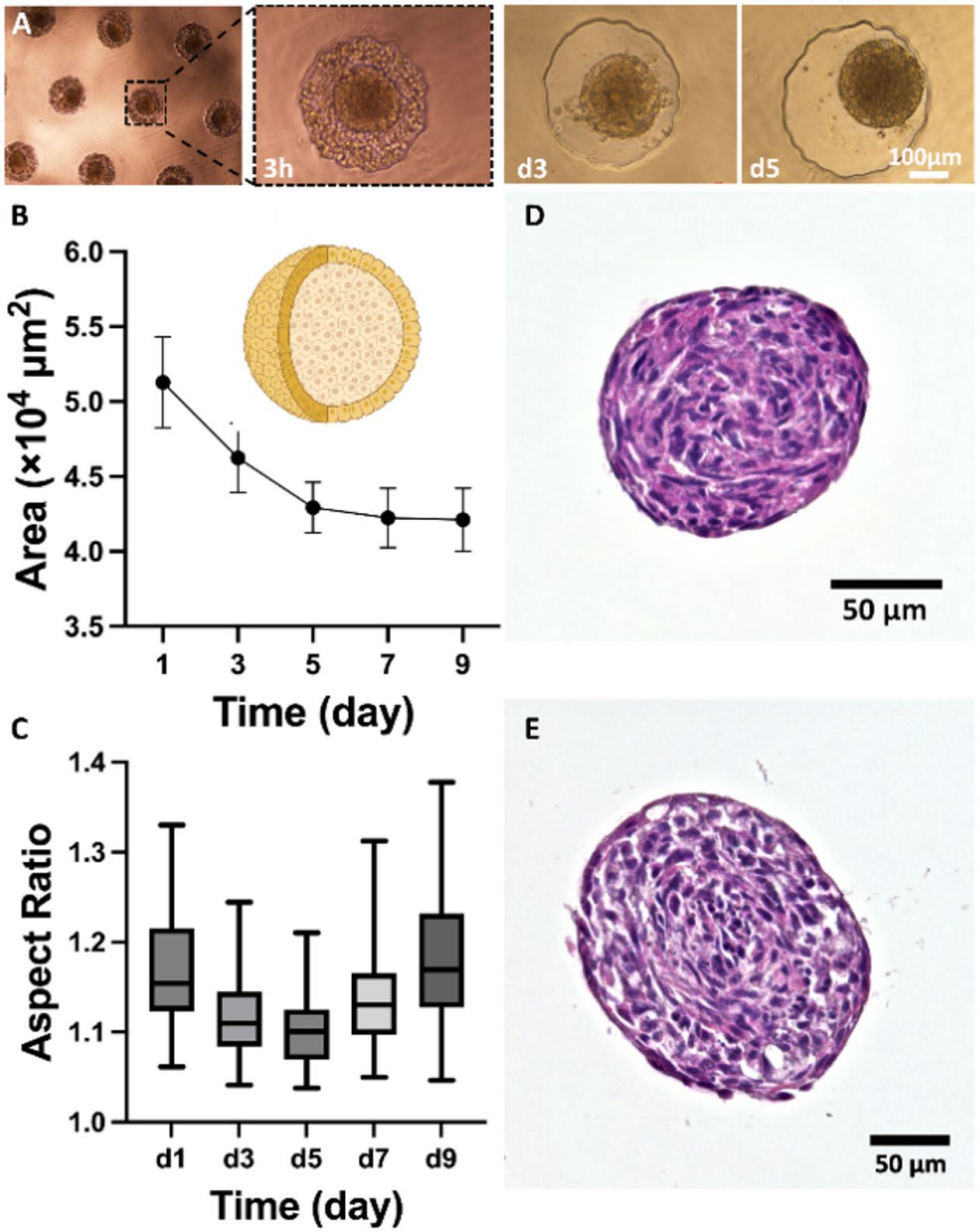
Formation and assessment of conjunctival spheroids (fibroblast-epithelial bilayer spheroids). (**A**) Optical images showing epithelial cells (1.5 K) progressively attach to fibroblast spheroids (3 K) within 3 h after seeding and form a stable bilayer by day 5. (**B**) Conjunctival spheroid area was quantified from brightfield images using ImageJ (*n* = 80). Mean ± SD areas were (5.1 ± 0.3) × 10^4^ μm^2^ at day 1, (4.6 ± 0.2) × 10^4^ μm^2^ at day 3, (4.3 ± 0.2) × 10^4^ μm^2^ at day 5, (4.2 ± 0.2) × 10^4^ μm^2^ at day 7 and (4.2 ± 0.2) × 10^4^ μm^2^ at day 9. (**C**) Mean ± SD aspect ratios (*n* = 80) were 1.168 ± 0.064 at day 1, 1.118 ± 0.041 at day 3, 1.104 ± 0.009 at day 5, 1.139 ± 0.024 at day 7, and 1.179 ± 0.034 at day 9. (**D**, **E**) Hematoxylin stains the nuclei of day 5 fibroblast spheroids (**D**) and day 5 conjunctival spheroids (**E**) blue-purple, while eosin stains the cytoplasm pink.

After developing 3 K fibroblast spheroids, 1.5 K primary conjunctival epithelial cells were seeded onto the spheroid surface on day 5 and incubated overnight on an orbital shaker to facilitate uniform adherence. Optical imaging revealed that epithelial cells began adhering within 3 h and progressively formed a continuous outer layer by day 3 and 5 (Fig. 4A). The epithelial coverage became increasingly compact and uniform over time, indicating successful integration with the fibroblast core. Quantitative area measurements further supported this observation (Fig. 4B). At day 1, spheroids exhibited a mean area of 5.1 ± 0.3 × 10^4^ μm², decreasing to 4.6 ± 0.2 × 10^4^ μm² at day 3 and 4.3 ± 0.2 × 10^4^ μm² at day 5, at this point the spheroids had largely stabilized in size. Minimal changes were observed thereafter, with sizes of (4.2 ± 0.2) × 10^4^ μm² at day 7 and (4.2 ± 0.2) × 10^4^ μm² at day 9, confirming that compaction and structural reorganization were essentially complete by day 5. Aspect ratio measurements remained close to 1 throughout the culture period (Fig. 4C). Mean aspect ratios were 1.16 ± 0.064 at day 1, 1.11 ± 0.041 at day 3, 1.10 ± 0.009 at day 5, 1.13 ± 0.024 at day 7, and 1.17 ± 0.034 at day 9, demonstrating consistent roundness and structural integrity of the bilayered conjunctival spheroids. In H&E-stained sections, fibroblast spheroids exhibited uniform nuclear and cytoplasmic distribution (Fig. 4D). In conjunctival spheroids, a homogeneous fibroblast matrix forms the core with a distinct boundary to the conjunctival epithelial layer (Fig. 4E).

**Fig. 5:**
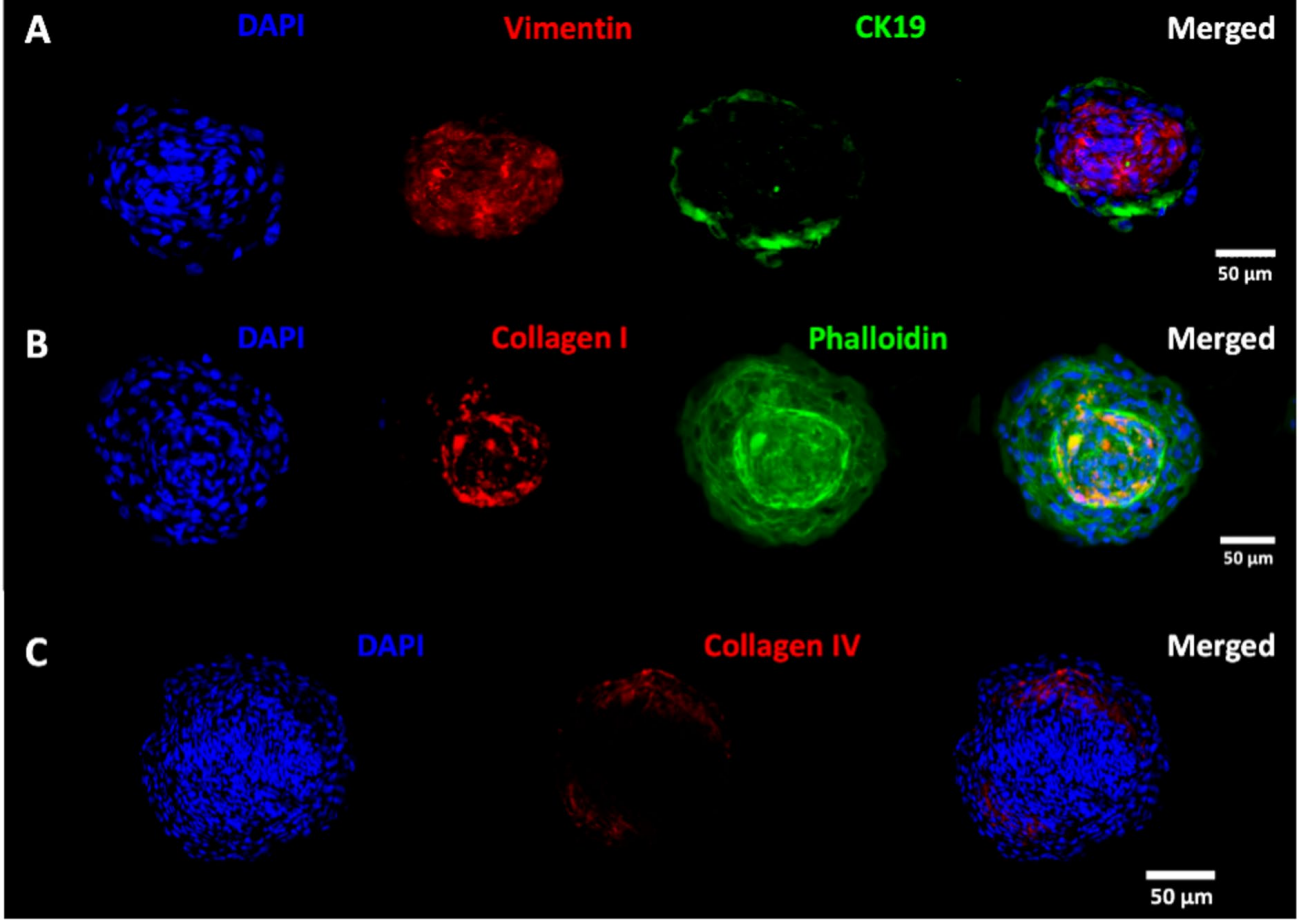
IF of conjunctival (day 10) spheroids. Nuclei were counterstained with DAPI (blue). (**A**) IF of conjunctival spheroids for CK19 (green), vimentin (red). (**B**) Collagen I is shown in red and phalloidin in green. (**C**) Collagen IV is shown in red.

To further characterize the cellular phenotype and extracellular matrix organization within the spheroids, immunostaining was performed for vimentin, CK19, collagen I, and collagen IV in day 10 conjunctival spheroids (Fig. 5). Vimentin was localized to the fibroblast stroma like core, whereas CK19, a conjunctival epithelial marker, was exclusively expressed in the outer epithelial layer (Fig. 5A). Collagen I immunoreactivity was confined to the central region, whereas the surrounding epithelial layer lacked collagen I signal, creating a distinct core-shell architecture consisting of a collagen I-rich core enclosed by an epithelial layer (Fig. 5B). By contrast, collagen IV delineated a basement membrane-like band at the interface between the fibroblast-rich core and the epithelial layer (Fig. 5C).

**Fig. 6:**
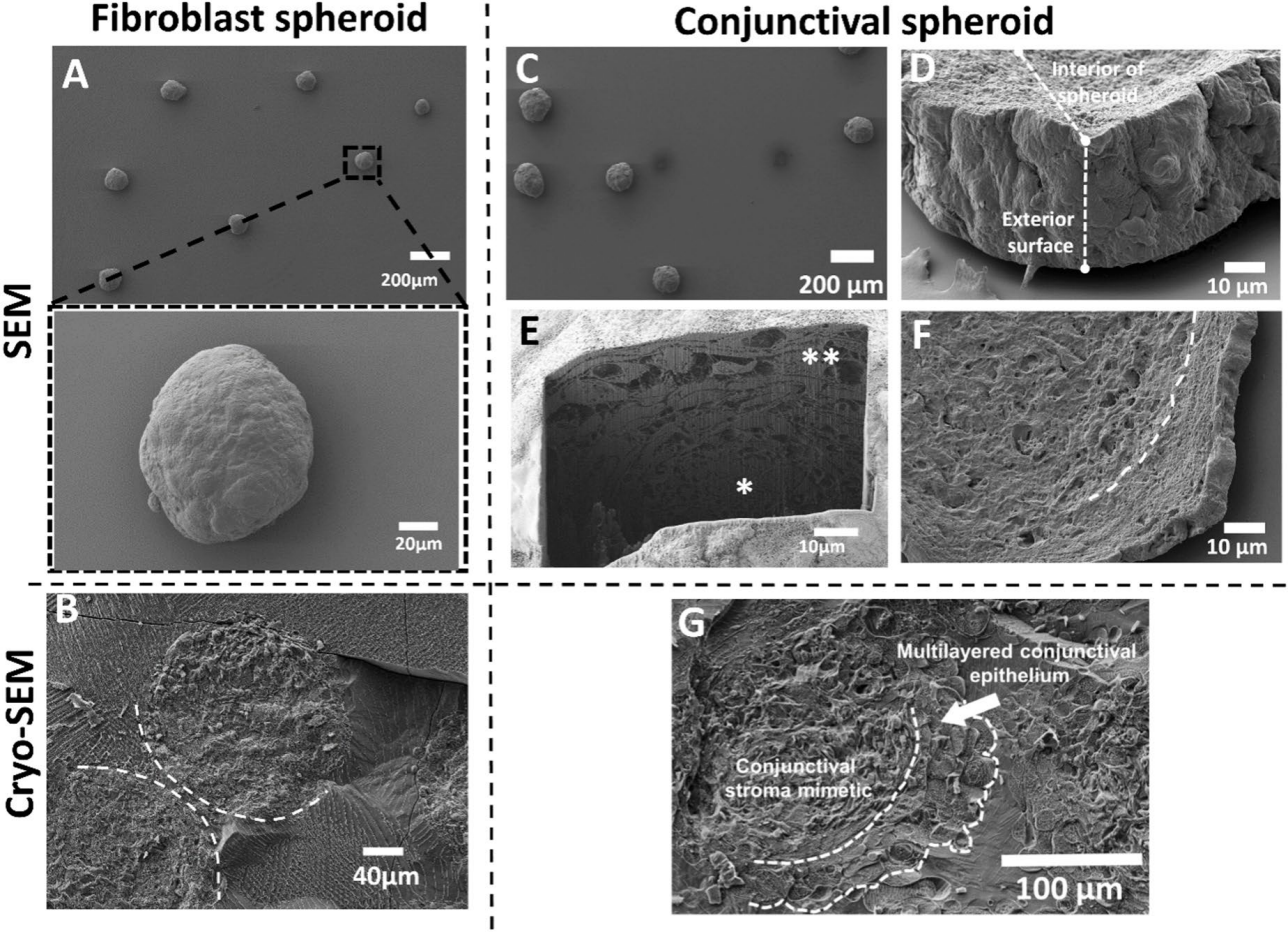
Scanning electron microscopy (SEM) and cryo-SEM images showing the morphology of fibroblast spheroids and bilayer conjunctival spheroids. (**A**) SEM and (**B**) cryo-SEM images of fibroblast spheroids demonstrating uniform spheroid size and well-organized stromal-like matrix. SEM of (**C**) intact and (**D**) cryo-sectioned conjunctival spheroid. (**E**) Focus ion beam-SEM of conjunctival spheroids, double star is representing less compact epithelial region and single star is representing more compact fibroblast region. (**F**) is representing SEM of cryosection of conjunctival spheroid. The dashed line indicates the approximate transition zone, defined based on differences in surface texture and tissue organization. (**G**) cryo-SEM image of conjunctival spheroid showing consistent overall morphology and clear bilayer architecture, with a multilayer epithelium enveloping a fibroblast-based stroma like core.

To further investigate the microarchitecture of the fibroblast spheroids and the bilayered conjunctival spheroids, SEM and cryo-SEM imaging were performed (Fig. 6). SEM imaging of day-5 fibroblast spheroids showed uniformly sized, compact spheres with smooth and continuous outer surfaces (Fig. 6A). Cryo-SEM provided additional insight into the internal stromal-like organization of these constructs, revealing densely packed fibroblasts and extracellular matrix components arranged in a cohesive three-dimensional network (Fig. 6B). Similarly, the SEM images of the conjunctival spheroids also revealed uniform surface (Fig. 6C). Higher-magnification SEM imaging of cryo-sectioned 50 μm-thick conjunctival spheroids further revealed a distinct epithelial exterior and a fibroblast-rich interior (Fig. 6D).

SEM imaging also showed the boundary between the epithelial layer and the fibroblast-derived stroma (Fig. 6F), though higher-resolution structural details were limited by the technique.

FIB-SEM cross-sectional imaging revealed heterogeneous ultrastructural organization within the bilayered conjunctival spheroids (Fig. 6E). The central region appeared relatively compact, exhibiting a dense morphology consistent with a fibroblast-rich core, whereas the lateral and peripheral regions showed a less uniformly compact structure, consistent with epithelial cell localization toward the spheroid surface. A gradual transition, rather than a sharp boundary, was observed between these regions. Irregular dark void-like features were present throughout the cross-section. These structures measured approximately 5–10 μm, comparable to nuclear dimensions. Based on their size and morphology, the voids are most consistent with nuclear or cytoplasmic material preferentially removed during focused ion beam milling, reflecting material pull-out typical of FIB-SEM analysis of soft biological specimens.

In contrast to SEM, Cryo-SEM, of the conjunctival spheroid revealed a bilayered tissue organization with distinct regional ultrastructural features (Fig. 6G). The inner region exhibited a dense, irregular, and fibrous morphology, characterized by a compact meshwork consistent with a stromal-mimetic compartment. In contrast, the outer region showed a multilayered cellular architecture, with flattened and tightly packed cells arranged parallel to the spheroid surface. A gradual morphological transition was observed between the inner stromal-like region and the outer epithelial compartment. While no sharp boundary was evident at the cellular level, clear differences in tissue texture, cell shape, and packing density distinguished the two regions.

**Fig. 7:**
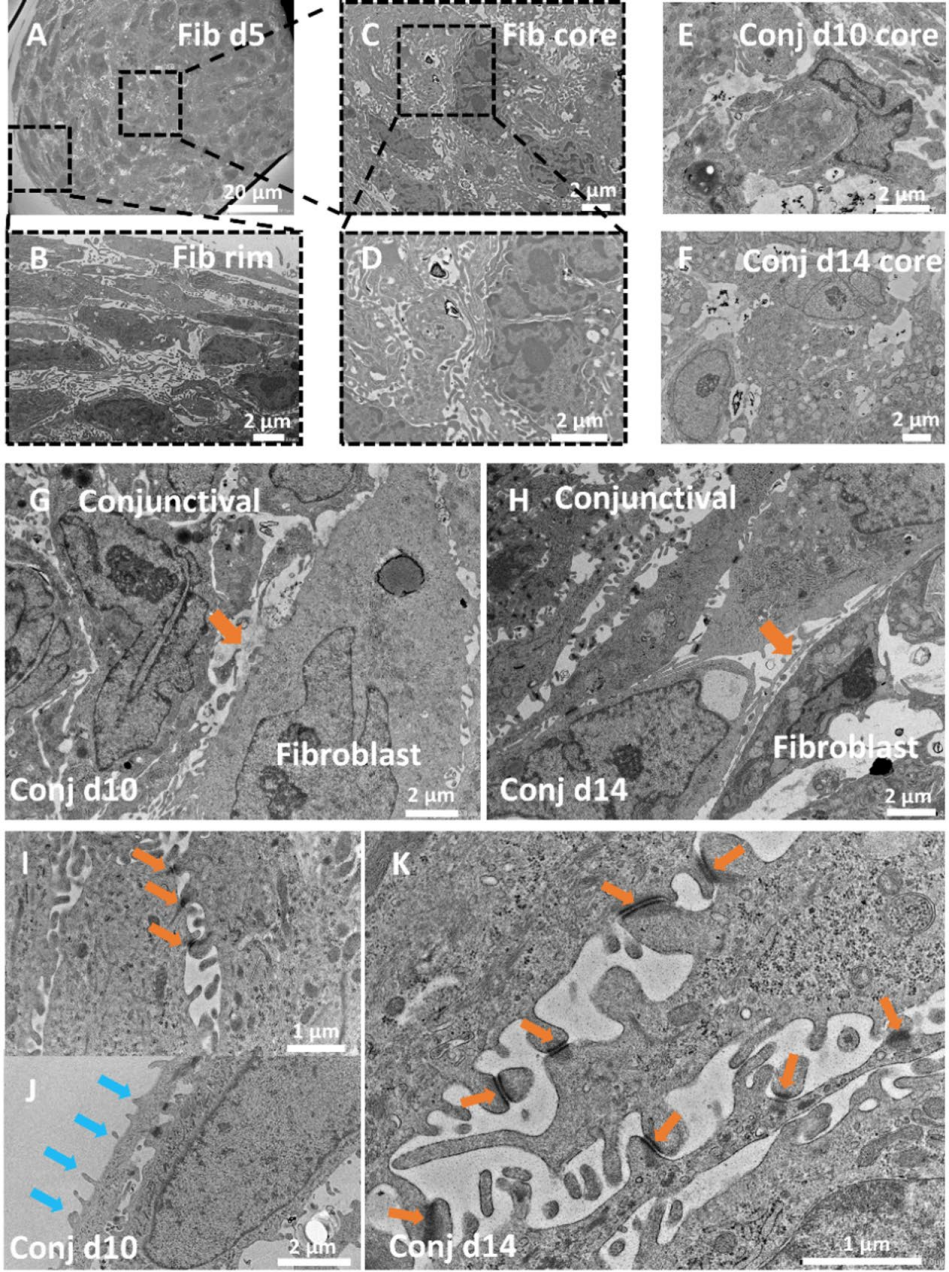
TEM images of fibroblast spheroids and conjunctival spheroids. (**A**–**D**) Fibroblast spheroid on day 5. (**B**) Higher-magnification view of the rim region, showing less compact ECM with more clearly defined fibrils and slightly larger intercellular spaces. (**C**, **D**) Core region, showing cells with intact plasma membranes and nuclei, extremely dense and lamellarly organized ECM, and filopodia-like extensions projecting from individual cells into the surrounding ECM. (**E**, **F**) Core region of conjunctival spheroids on days 10 and 14. (**E**) Core cells show swollen cytoplasm and vacuoles without overt necrosis; the ECM appears loosened and irregular. (**F**) The ECM is dense but interrupted by irregular voids and fragmented regions, and core cells appear atrophic. (**G**, **H**) Fibroblast-epithelial interface on days 10 and 14. (**G**) Conjunctival cells lie directly on irregular, fibrillar ECM (orange arrow). (**H**) A thin, irregular ECM layer separates the two cell types (orange arrow). (**I**, **J**) Epithelial cell contacts on day 10, showing close membrane apposition without mature junctions, with both direct contacts and ECM-separated regions (orange arrows); microplicae are present. (blue arrows) (**K**) Day 14, showing increased close membrane contacts (orange arrows).

In addition to SEM and cryo-SEM analyses, transmission electron microscopy (TEM) was performed to further assess the ultrastructural organization of the conjunctival spheroids. TEM confirmed the formation of bilayered conjunctival spheroids consisting of a fibroblast-based stromal compartment covered by a multilayered epithelial layer (Fig. 7). Pronounced ultrastructural differences were evident between fibroblast-only spheroids and bilayered constructs. At day 5, fibroblast-only spheroids exhibited a densely condensed architecture with abundant lamellar extracellular matrix (ECM). Fibroblasts retained an intact ultrastructural appearance despite the high degree of tissue compaction. Distinct cell-cell junctions were not observed, whereas filopodia-like extensions and membrane regions resembling focal adhesion sites were frequently detected. The ECM was more densely packed in the spheroid core, while the peripheral regions appeared less condensed, consistent with ongoing matrix remodeling at the surface. Following epithelial cell seeding, the stromal compartment underwent substantial ultrastructural changes. At day 10, fibroblasts displayed cytoplasmic swelling and vacuolization, and the surrounding ECM appeared less dense and more heterogeneous than in fibroblast-only spheroids. By day 14, fibroblasts remained detectable in the core but exhibited reduced cell volume and were associated with fragmented ECM. Conjunctival epithelial cells adhered to the fibroblast-derived stroma and formed an increasingly coherent outer layer between day 10 and day 14. Reduced intercellular spacing over time indicated progressive consolidation of the epithelial compartment. In addition, sparse and irregular apical membrane protrusions consistent with early microplicae-like structures were observed. However, a dense and continuous apical brush border was not evident. At the stromal-epithelial interface, epithelial cells were positioned directly on fibroblast-derived ECM. No continuous basement membrane was identified at either day 10 or day 14. At day 14, a thin and discontinuous interfacial matrix was present, suggesting early interface formation.

## Discussion

This study demonstrates the successful generation and scalable production of bilayered human conjunctival spheroids, providing a novel 3D in vitro test system for ocular surface research. The use of agarose-based microwell molds enabled precise control over spheroid size and architecture, resulting in high reproducibility and uniformity across experimental batches. In addition to their technical robustness, these molds are cost-effective, easy to implement, and do not require specialized infrastructure, thereby facilitating broader accessibility. In comparison to conventional agarose coating methods^26^(e.g., non-adherent agarose layers in culture plates), which often yield heterogeneous and poorly controlled spheroid formation, the use of defined microwell geometries allows for controlled cell aggregation, leading to more consistent spheroid size and cellular organization. This represents a significant improvement in standardization and experimental reproducibility. Compared to commercially available spheroid-forming platforms (such as ultra-low attachment (ULA) plates or proprietary microfabricated molds), the presented agarose-based system offers several advantages. Commercial systems, while user-friendly, are often associated with high costs, limited flexibility in design, and restricted scalability. In contrast, agarose molds can be fabricated in-house at minimal cost, allow customizable geometries, and can be readily adapted to specific experimental needs without dependence on proprietary formats. These features make the system particularly suitable for routine laboratory use and large-scale applications^27–32^.

Recently developed conjunctival organoid systems derived from epithelial stem/progenitor cells have demonstrated the capacity to recapitulate epithelial differentiation within extracellular matrix-dependent 3D cultures, typically requiring Matrigel-based scaffolds and extended culture periods of approximately 10–14 days to achieve functional maturation^15^. While these models are highly valuable for studying epithelial biology, they remain limited by their reliance on non-human, animal-derived matrices and the complete absence of a stromal compartment. In contrast, our study establishes a fully human, scaffold-free, and scalable 3D spheroid platform based on the co-culture of human primary conjunctival fibroblasts and epithelial cells. Our system enables rapid self-assembly into structurally stable bilayered spheroids within 48 h, driven by cell-mediated extracellular matrix deposition rather than exogenous materials. Importantly, the incorporation of fibroblasts facilitates the formation of a physiologically relevant stromal-epithelial interface, which is critical for tissue homeostasis and cell-cell signaling in the conjunctiva. This approach not only eliminates the variability and translational limitations associated with Matrigel-based systems but also provides a reproducible and high-throughput-compatible platform. Conceptually, this model represents a hybrid between organoid-based and tissue-engineered systems, combining the self-organization capacity of 3D cultures with a defined multicellular architecture that more closely reflects native conjunctival organization.

Live/dead staining confirmed high cell viability over time, supporting long-term spheroid maintenance. Minor cell death (in 4–5 K spheroids) is consistent with nutrient diffusion limitations in dense 3D structures and does not compromise overall tissue integrity. Cytoskeletal analysis revealed well-organized F-actin networks, essential for spheroid cohesion, and immunostaining confirmed lineage-specific marker expression, with CK19 in conjunctival spheroids and vimentin in fibroblast spheroids. These results validate the co-culture model as accurately recapitulating the epithelial and stromal components of native conjunctiva.

Histological evaluation with H&E staining further corroborated spheroid integrity and cellular organization. Fibroblast spheroids exhibited uniform, spindle-shaped cells, whereas conjunctival spheroids demonstrated a layered architecture with dense peripheral clustering, consistent with native tissue organization. Together, these findings confirm that the platform reliably generates bilayered conjunctival spheroids with structural and cellular fidelity suitable for physiological and disease modelling.

The cryo-SEM analysis provides structural evidence for successful formation of a bilayered conjunctival spheroid, comprising a stromal-mimetic core and an overlying multilayered epithelium^4^. The dense, fibrous organization of the inner region is consistent with fibroblast-rich conjunctival stroma, which in native tissue is characterized by abundant extracellular matrix and distinct cellular arrangement. The peripheral multilayered region exhibits key morphological features of stratified conjunctival epithelium, including flattened cells, tight packing, and organization parallel to the tissue surface. This architecture mirrors the non-keratinized, stratified epithelium of the native conjunctiva. Use of cryo-SEM minimized dehydration-related artifacts commonly associated with conventional SEM and avoided ion-beam-induced damage seen in FIB-SEM, enabling more faithful preservation of native-like tissue morphology^33^. TEM analysis demonstrated that the biofabricated spheroids reproduce several key ultrastructural features relevant to conjunctiva, including stromal self-assembly, epithelial attachment, early apical differentiation, and progressive bilayer formation^4,15,34^. The clear differences between fibroblast-only spheroids and bilayered constructs further indicate that epithelial-stromal crosstalk has a substantial impact on tissue architecture and stromal homeostasis^35^. In fibroblast-only spheroids, the dense lamellar ECM together with preserved fibroblast ultrastructure suggests that fibroblasts can tolerate pronounced mechanical confinement within a matrix-rich 3D environment. The absence of evident intercellular junctions, combined with the presence of filopodia-like processes and focal adhesion-like membrane specializations, supports the view that cohesion in these spheroids is mediated predominantly by ECM-dependent mechanical integration rather than by extensive direct cell-cell contacts. The lower matrix density at the periphery further points to active remodeling by surface-associated fibroblasts.

After epithelial cells addition, marked changes became apparent within the stromal compartment. Cytoplasmic swelling, vacuolization, and reduced ECM density at day 10 indicate that stromal behavior is altered by the presence of the epithelial cells. These findings are consistent with microenvironmental shifts induced by epithelial coverage, including changes in mechanical loading, paracrine communication, and diffusion conditions^4,15^. By day 14, the reduced fibroblast volume, matrix fragmentation in the spheroid core suggests declining stromal activity and the onset of nutrient and oxygen limitation, which are common challenges in larger avascular 3D culture systems^36,37^. The presence of sparse microplicae-like apical protrusions suggests partial initiation of superficial epithelial differentiation. However, the absence of a dense and continuous microplicae pattern indicates that apical maturation remained incomplete under the present culture conditions^38,39^.

Despite these advantages, the current model represents an early-stage tissue construct and does not yet fully recapitulate all features of native conjunctiva. In particular, the absence of goblet cells, a fully developed basement membrane, incomplete epithelial differentiation, and diffusion limitations in larger spheroids highlight areas requiring further optimization. In addition, the current system lacks immune and vascular components, and tear film which may play important roles in conjunctival physiology and disease. Consequently, the present model, in its current state should not be considered a comprehensive model of DED. Nevertheless, its bilayered epithelial-stromal organization makes it well suited for investigating epithelial-stromal interactions, inflammatory signaling, extracellular matrix remodeling, and barrier-related responses associated with ocular surface disease. Furthermore, the stromal compartment and scaffold-free architecture may provide opportunities for studying fibroblast activation and fibrotic conjunctival disorders, such as ocular cicatricial pemphigoid and trachoma, following further disease-specific validation.

In summary, these observations indicate that the current model represents an early developmental stage of conjunctival bilayer formation rather than a fully differentiated tissue equivalent. Further refinement of the culture system will be required to support more advanced tissue development. Extending the culture period, adjusting matrix composition, improving oxygen and nutrient delivery, and introducing differentiation-promoting conditions may facilitate basement membrane deposition and enhance epithelial differentiation.

## Conclusion and outlook

In this study, we established a conjunctival spheroid model as a novel 3D in vitro platform with substantial potential for disease modeling and translational research. By positioning this model as a primary human cell-based in vitro test system, the present work contributes to the development of animal-sparing model for ocular surface research. By applying advanced biofabrication strategies, we generated a reproducible and physiologically relevant system that recapitulates key aspects of native conjunctival tissue architecture, including epithelial-stromal organization and cell-cell interactions. The platform enables controlled and scalable production of bilayered spheroids with defined size, composition, and structural integrity. These features are particularly relevant for standardization, reproducibility, and transferability, which are key requirements for the broader implementation of NAMs in preclinical research.

The generated spheroids exhibited consistent morphology, narrow size distribution, and stable expression of characteristic conjunctival markers, confirming both structural fidelity and biological relevance. In contrast to conventional 2D cultures and epithelial-only models, the incorporation of both stromal and epithelial components provides a more representative microenvironment that more closely reflects in vivo tissue organization. Importantly, the system is entirely human-derived and scaffold-free, thereby avoiding the use of animal-derived matrices such as Matrigel and enhancing translational applications. This reduction in dependence on poorly defined animal-derived matrices may improve experimental reproducibility and supports the development of more human-relevant and ethically acceptable in vitro test systems.

The introduction of this spheroid-based platform represents a significant advancement in ocular surface research by enabling a more predictive and physiologically relevant model for investigating conjunctival (patho)physiology and therapeutic responses. In addition, its scalability and compatibility with high-throughput screening approaches position it as a promising tool for drug discovery and personalized medicine, while also aligning with the principles of reduction, refinement, and replacement (3R). More specifically, the model may contribute to reduction and partial replacement by providing a standardized human in vitro platform for early-stage compound screening, mechanistic studies, and disease-relevant testing before more complex or animal-based studies are considered.

At the current stage, the model does not yet recapitulate goblet cell differentiation, as spheroids were maintained under proliferative rather than differentiation-inducing conditions. Consequently, the system represents an early-stage conjunctival construct with limited secretory specialization. However, this limitation does not compromise its utility for studying epithelial-stromal interactions, inflammatory signaling, and early pathological processes of the ocular surface, which are central to DED and related disorders. Therefore, the model should be regarded as fit-for-purpose rather than as a complete reconstruction of the native conjunctiva. Its current context of use is most appropriate for investigating early epithelial-stromal responses, inflammatory activation, tissue organization, and preliminary therapeutic testing.

Future studies will focus on including goblet cell differentiation and further optimizing the biofabrication process to improve spheroid uniformity, maturation, and long-term stability. Approaches such as air-liquid interface culture, targeted biochemical stimulation, and dynamic culture systems are expected to promote epithelial maturation and functional specialization. In addition, integration with biomimetic hydrogels or microfluidic platforms may further enhance physiological relevance by enabling the simulation of key environmental factors associated with DED. The development of inflammation- or infection-based models within this system may further expand its applicability. Further validation using disease-relevant stimuli, reference compounds, donor-to-donor variability assessment, and comparison with established ocular surface models will be important to define the model’s predictive capacity and regulatory relevance.

In summary, this study establishes a scalable, fully human, and structurally relevant conjunctival spheroid platform that bridges the gap between reductionist epithelial models and complex tissue-engineered constructs. As such, it provides a robust foundation for next-generation in vitro ocular surface models and contributes to accelerating translational research in ophthalmology.

## Data Availability

The datasets generated during and/or analyzed during the current study are available from the corresponding author on reasonable request.
